# Hysteroscopic resection vs dilation and evacuation for treatment of caesarean scar pregnancy: study protocol for a randomised controlled trial

**DOI:** 10.52054/FVVO.14.1.008

**Published:** 2022-04-03

**Authors:** G Saccone, E Mastantuoni, C Ferrara, G Sglavo, B Zizolfi, M.C. De Angelis, A Di Spiezio Sardo

**Affiliations:** Department of Neuroscience, Reproductive Sciences and Dentistry, School of Medicine, University of Naples Federico II, Naples, Italy; Department of Public Health, School of Medicine, University of Naples Federico II, Naples, Italy

## Abstract

**Background:**

Caesarean scar pregnancy (CSP) is a type of ectopic pregnancy where the fertilised egg is implanted in the muscle or fibrous tissue of the scar after a previous caesarean section. Management options for women who opted for termination of CSP include sharp curettage, dilation and evacuation (D&E), excision of trophoblastic tissues, local or systemic administration of methotrexate, bilateral hypogastric artery ligation, and selective uterine artery embolisation with curettage and/or methotrexate administration. Recently hysteroscopic resection has also been proposed as an alternative option.

**Objective:**

To compare the surgical outcome of hysteroscopic resection with dilation and evacuation (D&E) for the treatment of caesarean scar pregnancy (CSP).

**Methods:**

Parallel-group, non-blinded, randomised clinical trial conducted at a single centre in Italy. Eligible women are those with singleton gestations at less than 9 weeks of gestation, and with thickness of myometrial layer ≥1 mm at the level of the ectopic. Inclusion criteria are women with CSP with positive embryonic/fetal heart activity who opted for termination of pregnancy. Patients will be randomised 1:1 to receive either hysteroscopic resection (i.e. intervention group) or D&E (i.e. control group). In both groups, 50 mg/m2 (based on DuBois formula for body surface area) of methotrexate (MTX) will be injected intramuscularly at the time of randomisation (day 1) and another dose at day 3. A third dose of MTX is planned in case of persistence of fetal heart activity on day 5. Participants will receive either D&E or hysteroscopic resection from 3 to 7 days after the last dose of MTX. A sample size of 54 women is planned.

**Main Outcome Measures:**

The primary outcome is the success rate of the treatment protocol, defined as no requirement for further treatment until complete resolution of the CSP as demonstrated by negative beta hCG levels and absence of residual gestational material on ultrasound examination..

**Study hypothesis:**

Hysteroscopic surgery is superior to D&E for the treatment of CSP.

**What is new?:**

The results of the trial will provide information on the best treatment for CSP.

## Introduction

Caesarean scar pregnancy (CSP) is a type of ectopic pregnancy where the fertilised egg is implanted in the muscle or fibrous tissue of the scar after a previous caesarean section (Timor-Tritsch et al., 2019). There are two types of CSP; CSP with growth towards the cervicoisthmic space or uterine cavity (type I, endogenic type) and those with deep invasion of scar defect and growth towards the bladder and abdominal cavity (type II, exogenic type) (Gonzalez et al., 2017).

Expectant management is acceptable in CSP with no fetal cardiac activity (Jayaram et al., 2018), while CSP with positive embryonic/fetal heart activity managed expectantly is associated with a high burden of maternal morbidity including severe haemorrhage, early uterine rupture, hysterectomy and severe placenta accreta, raising the question whether termination of pregnancy should be the treatment of choice for these women (Cali et al., 2018). Several approaches have been studied for the management of CSP in women who opted for termination of pregnancy, including sharp curettage, dilation and evacuation (D&E), excision of trophoblastic tissues, local or systemic administration of methotrexate, bilateral hypogastric artery ligation, and selective uterine artery embolisation with curettage and/ or methotrexate administration (Karahasanoglu et al., 2018). Recently, operative hysteroscopy with resection, has been proposed as a safe and effective alternative for the treatment of CSP (Qian et al., 2015; Fylstra, 2014). Operative hysteroscopy has many advantages compared to the traditional blind approach and has continued to grow for management of different conditions, including retained products of conception (Alonso Pacheco et al., 2019), or incomplete abortion (Golan et al., 1992).

## Objective

The aim of this trial is to compare the success rate of hysteroscopic resection with D&E for the treatment of CSP.

## Methods

### Study design and participants

This is a single-centre parallel group randomised controlled trial (RCT) of women with CSP who opt to have termination of pregnancy using either hysteroscopic resection or D&E at the University of Naples Federico II (Naples, Italy).

The trial was approved by the local ethics committee. All participants in the trial provided written informed consent.

Eligible women are those who opt to have termination of pregnancy with singleton gestations of less than 9 weeks gestation with positive embryonic/ fetal heart activity and with ≥1 mm myometrial thickness overlying the pregnancy . Both type I and type II CSP are included. Exclusion criteria are multiple gestations, heterotopic pregnancy, diagnosis of cervical pregnancy, failing intrauterine pregnancy or any other anomalous implantation site, negative fetal heart activity at the time of randomisation and myometrial thickness of <1 mm.

### Randomisation and masking

Eligible participants will be randomised 1:1 to receive either hysteroscopic resection (i.e. intervention group) or ultrasound-guided D&E (i.e. control group). Randomisation will be via a web- based system that is prepared by an independent statistician. The recruiters and the trial coordinator do not have access to the randomisation sequence. The trial is open label but the data analysts will be blinded to allocated treatment group.

### Interventions

50 mg/m2 (based on DuBois formula for body surface area) of methotrexate (MTX) (Du Bois and Du Bois, 1989; Kutuk et al., 2014) will be injected intramuscularly to all participants in both groups at the time of randomisation (day 1) and another dose on day 3. A third dose of MTX is planned in case of persistence of positive fetal heart activity on day 5.

Women in the control group will receive D&E from 1 to 5 days after the last dose of MTX. D&E is performed under ultrasound guidance under spinal anaesthesia. A speculum is placed in the vagina to see the cervix and a tenaculum is placed to steady the cervix. The cervix is dilated using Hegar dilators. After sufficient dilation, sharp curettage with or without suction evacuation with Karman cannula, is performed (Stubblefield 1986).

Women in the intervention group will receive hysteroscopic resection from 1 to 5 days after the last dose of MTX. Hysteroscopic resection will be performed under spinal anaesthesia (Giampaolino et al. 2018), using 15 Fr bipolar miniresectoscope. All included women are admitted for inpatient management.

### Diagnosis of caesarean scar pregnancy

Diagnosis of CSP is made by transvaginal ultrasound using the following criteria as described by Timor-Tritsch et al. (2012); visualisation of an empty uterine cavity and empty endocervical canal; detection of the placenta and/or gestational sac embedded in the hysterotomy scar; triangular gestational sac that fills the niche of the scar; a thin myometrial layer (1-3 mm) between the gestational sac and the bladder; a closed and empty cervical canal; presence of embryonic/fetal pole; presence of a prominent and at times rich vascular pattern at or in the area of the caesarean scar in the presence of a positive pregnancy test.

### Outcomes

The primary outcome is the success rate of the treatment protocol, defined as no requirement for further treatment until complete resolution of the CSP as demonstrated by negative beta hCG levels and absence of residual gestational material on ultrasound examination. Treatment failure is defined as the need for further treatment before complete resolution of the CSP. Secondary outcomes are use of additional procedures, intra- and postoperative complications, blood transfusions, admission to intensive care unit (ICU), maternal death and length of stay (LOS) in the hospital.

### Sample size calculation

The sample size calculation was based on detecting an effect that produces an increase in the primary outcome from a baseline successful rate of 65% (women undergoing D&E) (Kanat-Pektas et al., 2016) to a success rate of 95% (women undergoing hysteroscopy surgery) (Ash et al., 2007; Deans and Abbott, 2010; Birch Petersen et al., 2016). Given an alfa of 0.05 and 80% power, a sample size of 54 women is planned (27 for each intervention group).

### Statistical analysis

Univariate comparisons of dichotomous data will be performed with the use of the chi-square test with continuity correction. Comparisons between groups will be performed with the use of the T-test to test group means by assuming equal within- group variances.

The primary analysis will be an intention to treat comparison of the treatment assigned at randomization. The effect of hysteroscopic resection on the cumulative incidence of each outcome will be quantified as the relative risk (RR) and its 95% confidence interval (CI). A 2-sided P value less than .05 was considered significant.

Statistical analysis was performed using Statistical Package for Social Sciences (SPSS) v. 19.0 (IBM Inc).

## Discussion

CSP is a rare form of ectopic pregnancy with the incidence raising globally. CSP is associated with high risk of maternal morbidity and potential mortality. Recently, a review by Birch Petersen et al. (2016) evaluated treatment modalities for CSP, focusing on efficacy and complications. They found limited data on the superiority of any intervention over others, with recommendations primarily based on case series and retrospective studies.

Only 4 randomised trials (Peng et al. 2015; Zhuang and Huang 2009; Qian et al. 2015; Li et al. 2011) evaluating treatment of CSP have been published so far. All of them were performed in China. Zhuang and Huang (2009) compared uterine artery embolisation (UAE) with systemic MTX (sample size 72 patients). Li et al. (2011) compared systemic MTX with transcatheter arterial chemoembolisation using different embolic agents (sample size 26 patients). Peng et al. (2015) compared local with systemic MTX (sample size 104 patients). Finally, Qian et al. (2015) compared D&C with operative hysteroscopy in combination with preventive UAE (sample size 66 patients). Notably, UAE appears as a technique associated with a high risk of major complications (Kröncke and David 2019).

To the best of our knowledge, this is the first randomised trial comparing the success rates of hysteroscopic resection with D&E for treatment of CSP following MTX treatment. We anticipate that hysteroscopic surgery has beneficial effects on treatment of CSP compared to D&E. The results of the trial will provide information on the best treatment for CSP.
